# *Candidozyma auris* (*Candida auris*) as a harbinger of novel fungal pathogens

**DOI:** 10.1128/msphere.00109-26

**Published:** 2026-10-02

**Authors:** Carla E. Schuh, Chaitanya S. Gokhale

**Affiliations:** 1 Chair for Computational and Theoretical Biology, University of Würzburg 9190, Würzburg, Germany; 2 Joint Institute for Individualisation in a Changing Environment (JICE), University of Würzburg 9190, Würzburg, Germany; Shenzhen Institute of Synthetic Biology, Shenzhen, China

**Keywords:** antifungal resistance, thermotolerance, adaptive immunity, climate change

## Abstract

Mammals are protected from fungal pathogens by two evolutionary pillars: adaptive immunity and elevated body temperature. Anthropogenic climate change is eroding this thermal barrier, while a concurrent decline in human basal body temperature accelerates convergence from both directions. *Candida auris*, a thermotolerant, multidrug-resistant pathogen, arose independently as genetically distinct clades on multiple continents, a pattern implicating shared selective pressures rather than clonal spread. Unlike its closest relatives, which grow poorly at mammalian temperatures, it thrives at 37°C, tolerates up to 47°C, persists on hospital surfaces, resists all three major antifungal classes, and causes invasive infections with 30–60% mortality. We review its evolutionary biology, ecology, and clinical significance, evaluating hypotheses for its origin, and the mechanisms underlying its thermotolerance and virulence. *C. auris* may be the first of many fungi adapting to breach mammalian thermal defenses, making predictive frameworks for fungal disease emergence an urgent priority.

## INTRODUCTION

For most of mammalian evolutionary history, fungi posed a negligible threat to mammals. While devastating to plants and ectothermic animals, and causing agricultural catastrophes, fungal pathogens were largely unable to breach the defenses of warm-blooded hosts (1). With a core temperature of approximately 37°C against a global mean temperature of roughly 15°C, mammals maintain a thermal gradient that only a fraction of the estimated 3–5 million fungal species can overcome (2, 3). A highly efficient adaptive immune system serves as a second line of defense. With these “twin pillars of protection” intact, mammals remain remarkably resistant to fungal disease (4). Of the approximately 148,000 described fungal species, only 300–400 (0.3–0.4%) are currently capable of infecting humans, and most of these are opportunistic pathogens that primarily threaten immunocompromised individuals (5).

This thermal barrier, however, is eroding. With a current gap between global mean temperature (15.08 ± 0.5°C) (6) and average human body temperature (37°C) of approximately 22°C, every 1°C rise in global mean temperature reduces the protective thermal gradient by approximately 5% (7). In environmental fungi, climate change is thought to be selecting for thermotolerance (7), already evident among basidiomycetes (8). Compounding this pressure is a decline in human basal body temperature over the past century (9). The convergence of warming environments and cooling hosts accelerates the collapse of the millennia-old thermal shield of mammals.

Consequences of this erosion may already be manifesting. *C. auris* may represent the vanguard of a new era in infectious disease. Within a decade of its identification in Japan in 2009 (10), *Candida auris* emerged on five continents as genetically distinct, multidrug-resistant clades, each thriving at mammalian body temperature where its closest relatives grow poorly (3, 11). This simultaneous, independent emergence is unprecedented in medical mycology and points to a common selective pressure, plausibly rooted in global environmental change.

Historically, fewer than 0.1% of described fungal species could grow above ambient temperature (7). If climate change enables even a small fraction of environmental fungi to breach the thermal barrier, the implications are profound: mammals inhale 1–10 fungal spores with every breath, and fungi constitute the majority of airborne biomass (12), making exposure unavoidable. Already, at least 6.5 million people suffer from invasive fungal infections each year, with 2.5 million deaths directly attributable. These figures are almost certainly underestimates given uneven global surveillance and diagnostics (13). Our current knowledge about fungal evolution and phylogeny is severely limited (14), mainly due to a lack of fungal fossil records and the broad diversity within the kingdom regarding morphology, lifestyles, genomic plasticity, and more (14).

With the taxonomy of the polyphyletic *Candida* genus still largely unresolved and many of its members only distantly related, the correct classification is a subject of ongoing debate and research, further complicating the identification and documentation of *C. auris* cases (15). Recent reclassifications of several members into the genus *Candidozyma* (15–17), including *Candida auris* (now *Candidozyma auris*), aim to better reflect their distant relation to *Candida* spp., such as *Candida albicans* and *Candida parapsilosis* (16). For continuity of nomenclature, *Candida auris* will be used throughout this review.

**Fig 1:**
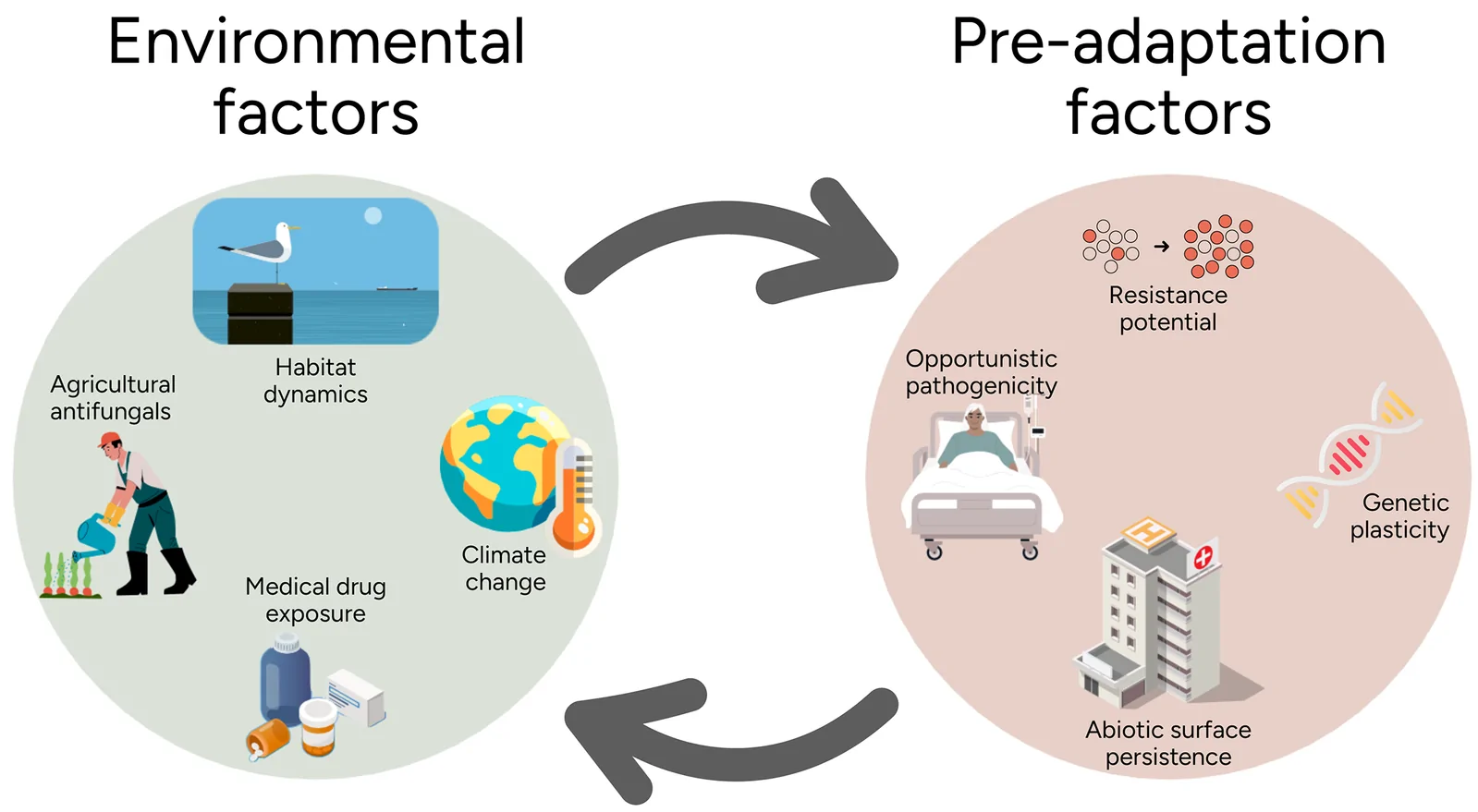
A visual summary of the hypothesized environmental influences that made *C. auris* into the pathogen we observe today. Factors in its natural environment (competition in a dynamic habitat, climate change, drug exposure in the medical context, and agricultural antifungals, green circle) shaped a species with pre-adaptations (resistance potential to antifungals, genomic plasticity, ability to persist in hospitals, and being an opportunistic pathogen, brown circle). These factors influence one another, creating a feedback loop that produces a highly competent fungal pathogen with unprecedented characteristics.

This review examines *C. auris* as a case study for understanding how climate change may fundamentally alter the landscape of human fungal disease. We explore its multi-continental emergence and discuss mechanisms of its rise, from molecular virulence factors to ecological hypotheses and consider what it reveals about our vulnerability to future fungal threats. Unlike bacterial and viral pathogens, fungi have remained largely relegated to tropical medicine and immunocompromised populations. *C. auris* challenges this complacency, demonstrating that environmental fungi can rapidly evolve to cause severe disease while simultaneously acquiring resistance to our limited antifungal arsenal. Understanding interwoven evolutionary and ecological forces that shaped *C. auris*, as visually summarized in Fig. 1, is therefore essential preparation for a future in which the mammalian thermal barrier continues to erode.

## THE *C. AURIS* CASE STUDY: EMERGENCE, SPREAD, AND CHARACTERISTICS

### Emergence and spread

In 2009 in Japan, an unusual yeast was isolated from a patient’s ear canal and designated *Candida auris* (10). Retroactive analysis revealed that the organism had been circulating undetected since at least 1996 in South Korea (18). This delayed identification reflects a broader problem in clinical mycology. Phenotypic methods are slow and unreliable for distinguishing closely related species, and *C. auris* was absent from diagnostic databases for years after its description (19), often misidentified as *C. haemulonii* or related species (20–22). The problem is compounded in regions lacking diagnostic infrastructure: sub-Saharan Africa, South America, and Eastern Europe (11, 23, 24). With adequate surveillance, the picture is striking. In Kenya, *C. auris* accounts for 38% of candidemia cases (11), suggesting the true global burden is substantially higher than current estimates. By 2016, medical institutions worldwide were formally alerted to *C. auris* as an emerging pathogen. By 2019, it had been recovered from over 4,000 blood isolates across 40 countries (11, 25). From 2017 to 2023, reported case numbers increased two- to fivefold (26–29).

**Fig 2:**
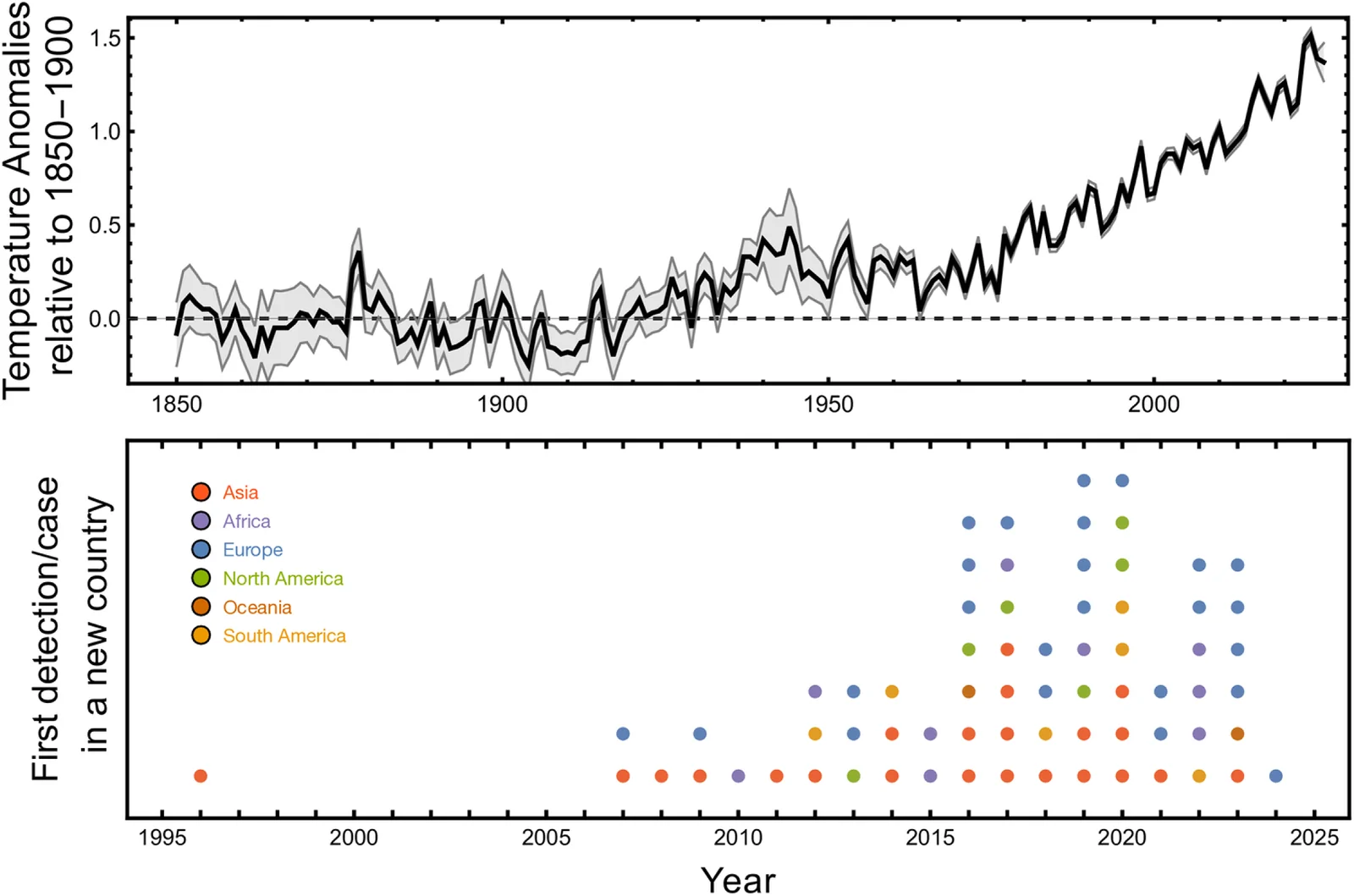
Plots of the global temperature anomalies and the first reported cases in countries classified per continent over time. The top panel depicts mean temperature anomalies, in an uncertainty band, relative to the reference period 1850–1900 (32). As per the Intergovernmental Panel on Climate Change (IPCC) standard (33), temperature anomalies are plotted rather than mean temperature, as the margin of uncertainty is lower for these data sets. Data set anomalies are calculated relative to a 1981 to 2010 baseline and offset by 0.69°C, which is the best estimate of the difference for that period from the 1850–1900 average, as given in the IPCC Sixth Assessment Report. Temperature anomalies reflect the difference between current temperatures relative to an averaged historical baseline. In the bottom panel, each dot represents the earliest known sample of *C. auris* in a new country, either in the form of retroactively identified, historic isolates or new medical cases. They are sorted by the year the sample was taken, not by when it was discovered. The dots are color-coded by continent. Multiple dots of the same color in a year indicate detections in different countries within the same continent.

*C. auris* presents a pattern of emergence unprecedented in medical mycology. Between 2009 and 2016, four major clades appeared independently in distinct geographic regions (see Fig. 2), each exhibiting unique genetic signatures and resistance profiles (11, 26), with two additional clades reported in the last decade (30, 31). Despite sharing high sequence identity, these clades exhibit 17-fold greater genetic diversity between them than within them, confirming independent evolutionary trajectories (11, 25). Molecular clock estimates place the emergence of individual clades in the late 1990s to early 2000s, their most recent common ancestor predating industrial-era warming (25).

A transition appears to have occurred between 2013 and 2015 (see Fig. 2). *C. auris* cases moved from sporadic cases to prolonged nosocomial (hospital) outbreaks (22). This may be attributed to increased awareness and sampling efforts or genuine changes in transmission and epidemiological dynamics where *C. auris* might have crossed a certain threshold that drove its spread and increased detections.

Molecular epidemiology reveals clusters of highly related isolates within facilities, indicating sustained local transmission. Yet, multiple countries harbor isolates from different clades, demonstrating repeated independent introductions (25). Incomplete travel data complicates transmission tracking: in the United States, only 7% of cases had documented healthcare exposure abroad, despite isolates spanning multiple continental clades (25). This circumstance most plausibly reflects unrecognized colonization among transferred patients and healthcare workers, though cryptic environmental reservoirs cannot be excluded. Furthermore, the six clades show distinct profiles of antifungal resistance, virulence, and phenotype, as reviewed in detail here (11, 25, 34). *C. auris* seems to have efficiently exploited the human travel pathways and trade networks (35–37).

### Characteristics

*C. auris* has become a major opportunistic pathogen. Bloodstream infections carry a mortality of 20–67%, and nearly 52% in most countries (38, 39); in one European outbreak of 140 cases, 41% died within a month (40). Immunocompromised patients are at the highest risk, though infections in immunocompetent individuals have been documented (41–43). Risk factors mirror those for other invasive Candida infections: immunosuppression, indwelling catheters, prior antimicrobial exposure, and prolonged ICU stays (44–46). Early presentation is non-specific, and blood cultures are often negative at first, delaying diagnosis (40); survival improves sharply with intervention within 12 h that delayed identification routinely costs (47). We highlight three phenotypic traits underlying this clinical profile before discussing their mechanistic basis.

#### Thermotolerance

The most striking trait, and the one central to the climate-emergence hypothesis, is exceptional thermotolerance. *C. auris* thrives at 37°C, some isolates surviving up to 47°C, whereas its closest relatives grow poorly in this range (3, 48). This capacity exceeds that of most fungi and many established human pathogens.

#### Resistance

Current guidelines recommend azoles or echinocandins for prophylaxis and treatment (49). *C. auris* exhibits resistance to all three major antifungal classes: up to 90% of isolates are resistant to azoles, 30% to amphotericin B, and 2–8% to echinocandins (26, 50, 51), with pan-resistant isolates documented (50). As fungi and humans are both eukaryotes, potential drug targets are inherently limited (52).

#### Persistence

*C. auris* remains viable on hospital surfaces for months and withstands standardized cleaning and disinfection (39, 53), underpinning its shift from sporadic infection to persistent nosocomial outbreaks. Transmission combines environmental contamination and patient-to-patient spread in proportions that vary across outbreaks and involves both symptomatic patients and asymptomatic carriers (11, 54). Whether it can enter a viable-but-non-culturable state, as some fungal pathogens do, remains unresolved (11).

## MECHANISTIC UNDERSTANDING OF THE SUCCESS OF *C. AURIS*

The near-simultaneous, multi-continental emergence of *C. auris* poses a fundamental question (Fig. 2): how did this organism successfully breach mammalian defenses and spread globally when millions of other fungal species have not? The answer lies in the convergence of the three traits described above, not in a single evolutionary innovation. Critically, these traits arise from conserved fungal stress response systems that may be latent in many environmental species, awaiting the appropriate selective pressures to be activated.

### Thermotolerance foundation

The thermotolerance of *C. auris* derives not from novel molecular innovations but stress-response pathways widespread across fungi (3). Current evidence gives no indication that these pathways function fundamentally differently in *C. auris* than in other fungi. Thermal stress endurance capacity is latent in many lineages, and its activation under climate-driven selection is anticipatable rather than idiosyncratic. Central among these is heat shock protein 90 (HSP90), an essential molecular chaperone that stabilizes proteins involved in stress responses and is upregulated under thermal stress across diverse fungal taxa (55–57). While further research is needed on this topic, current findings suggest that the exact molecular mechanisms of HSP90 across species vary but result in enhanced stress tolerance regardless (58). In *C. auris*, HSP90 depletion triggers morphological changes and compromises drug tolerance (39, 57). Some lineages developed HSP90-independent drug resistance (57) through fixed mutations, suggesting an evolutionary trajectory consistent with a model in which HSP90 initially buffers (thermal) stress long enough to allow for the accumulation of adaptive genetic changes, which eventually render the chaperone dispensable (59). The exact mechanism remains unclear, but mutations affecting ERG11, specifically the Y132F variation, CDR1, TAC1, FUR1, FCY2, and FKS1 are thought to play a role in the emergence of HSP90-independent drug resistance (51, 57, 59–64). Current research suggests that epimutations and epigenetic pathways confer drug tolerance in diverse fungi (65–68). It is possible that similar mechanisms shape the seemingly strain-specific resistance profiles of *C. auris* (69), but this remains to be investigated further.

Additional protection is provided by Hog1, a stress-activated protein kinase with pleiotropic roles in osmotic and thermal stress responses. Deletion of Hog1 reduces virulence and alters cell morphology, underscoring its importance for pathogenesis (70, 71), with strain-specific results (71). That HSP90 and Hog1 are evolutionarily conserved across fungal taxa is a key point: the molecular machinery for thermotolerance exists widely and does not need to evolve *de novo*.

With shared machinery, why did *C. auris* emerge as a pathogen when its relatives did not? The distinction lies not in conserved pathways but in the genomic and phenotypic context in which they operate. *C. auris*’s lineage-specific features cannot be captured by a stress-response account alone: copy-number variation and expanded families of drug transporters and lipases (72), clade-specific chromosomal rearrangements and a variable subtelomeric repertoire of cell-surface adhesins (73), clade-specific amplification of the azole target *ERG11* (25), and reversible, high-frequency phenotypic switching that generates standing variation in virulence traits (74). These are not conserved stress machinery but contingent products of this lineage’s genome architecture and plasticity. We therefore separate two easily conflated claims. The conserved machinery explains why the thermal barrier is crossable, and its eventual crossing is predictable across fungi.

### Multidrug resistance as cross-protection from environmental stress

Multidrug resistance is facilitated through mutations in drug targets, altered ergosterol biosynthesis, amplification of efflux pumps, the conserved carbonic anhydrase gene NCE103, and pyruvate-regulating genes (57, 60, 69, 75–78), with varying profiles between clades and isolates (77, 79). Particularly alarming is the speed at which this resistance evolves: experimental populations developed high-level echinocandin resistance within three days (80), raising concerns that novel antifungals may provide only a temporary reprieve.

A more fundamental insight concerns *C. auris*’s origins of resistance. It demonstrates tolerance to reactive oxygen species, high salinity, and cell wall stress, a profile consistent with extreme environmental niches (3, 70). Fungal stress responses are often cross-protective: genes upregulated in response to one stressor commonly confer resistance to seemingly unrelated challenges (52). Selection for environmental stress tolerance may thus inadvertently co-select for drug resistance, explaining how *C. auris* arrived in clinical settings already equipped with multidrug resistance despite minimal prior exposure to antifungal agents.

This cross-protection hypothesis is reinforced by the elevated ergosterol and structural lipid content of *C. auris*, which simultaneously enhances membrane stability under environmental stress and reduces antifungal efficacy (11). The same HSP90 system that enables thermotolerance also facilitates drug tolerance by stabilizing stress-response proteins and promoting rapid phenotypic plasticity (57). In other words, the mechanisms are not independent but facets of an integrated stress response architecture.

### Persistence: the hospital reservoir

Persistence rests on two interconnected strategies: biofilm formation and specialized survival structures.

The third component of *C. auris*’s success is its capacity for prolonged environmental persistence, which, despite adherence to standardized hygiene protocols, enabled it to claim hospitals as a novel reservoir (11, 39). This persistence stems from two interconnected strategies: biofilm formation and specialized survival structures.

Biofilms are the predominant mode of microbial life in natural environments (81). In general, viable fungal cells or spores need to reach a suitable surface and attach to it, followed by rapid replication and expansion, enabling microcolony formation. As the microcolonies mature, they form a biofilm with an increasingly complex 3D structure. Finally, the fungus disperses into the environment. In *C. auris*, it is known that biofilms composed of blastoconidia and pseudohyphae are capable of reducing drug susceptibility through physical barriers and cellular aggregation (39). Adhesion is conferred by adhesins Als4112 and Scf1 (82, 83), which are thought to enable aggregation as well, though the latter has only been observed in certain strains (84). Aggregation is associated with enhanced biofilm formation capabilities (84–87). Most concerning is *C. auris*’s ability to colonize surfaces 4 h after initial contamination (88).

In *C. auris*, it is known that biofilms composed of blastoconidia and pseudohyphae are capable of reducing drug susceptibility through physical barriers and cellular aggregation (39). Adhesion is conferred by adhesins Als4112 and Scf1 (82, 83), which are thought to enable aggregation as well, though the latter has only been observed in certain strains (84). Aggregation is associated with enhanced biofilm formation capabilities (84–87). Most concerning is *C. auris*’s ability to colonize surfaces 4 h after initial contamination (88).

As a second persistence strategy, *C. auris* has been observed to form giant lipid droplets (gLDs) under nutrient-poor conditions (53). gLDs enhance resistance to environmental, thermal, and antifungal stress, linking stress tolerance to nutrient storage through a single mechanism.

Environmental fungi experience low-to-moderate but constant stress. Their survival systems have evolved accordingly (52). Laboratory conditions typically involve glucose-rich growth media and acute, high-dose stress exposures. What appears as virulence or drug resistance in clinical settings may therefore represent pre-existing adaptations.

### Pre-adaptations rather than novel evolution

The mechanistic picture is not a recent evolutionary innovation but the activation of a pre-existing adaptive toolkit, one that evolved in stressful environments rather than in human pathogenesis (3, 11). Climate change may not have created a pathogen *de novo* but rather shifted environmental conditions such that fungi with certain pre-existing traits suddenly found mammalian body temperature within their thermal tolerance range—a prime example of ecological opportunity. More plausibly, agricultural antifungals amplified resistance mechanisms already selected through cross-protection, coincidentally facilitating pathogenesis.

These mechanisms are not unique to *C. auris*. Robert et al. proposed that thermotolerance was common among ancestral fungi and was lost in many lineages after a global cooling of approximately 5°C some 34 million years ago (89, 90). If so, its genetic architecture is more broadly conserved than currently appreciated and could re-emerge under sustained warming. Fungi also carry an enzymatic arsenal for degrading organic substrates that contributes to virulence in host contexts (91). Identifying where these pressures act next is critical for predicting which species follow.

## EVOLUTIONARY AND ECOLOGICAL CONTEXT

If the molecular toolkit for thermotolerance and stress resistance is widespread across fungi, why did *C. auris* emerge now? What does its emergence pattern reveal about selective pressures currently acting on environmental fungi worldwide?

### The missing reservoir

Despite intense investigation, the environmental reservoir of *C. auris* remains unknown. Its closest phylogenetic relatives inhabit remarkably disparate ecological niches: flowers, human microbiomes, or the gastrointestinal tracts of marine animals (11). Phylogenetic analysis confirms broad environmental flexibility. The interspersion of environmental and clinical isolates among close relatives suggests previous transitions between free-living and host-associated states (92). However, the substantial genetic distance separating *C. auris* from these relatives limits the utility of phylogenetic inference for reconstructing its ancestral ecology.

The lack of population genetic data from ancestral populations further constrains our understanding of the evolutionary trajectory (11). Comparative genomics reveals changes in genes associated with drug resistance and virulence, suggesting positive selection, but these analyses remain inconclusive without knowledge of ancestral population parameters (11). Discovery and sequencing of more closely related species could illuminate *C. auris*’s evolutionary history, but they remain elusive.

Physiology offers partial clues. *C. auris* tolerates high salinity (10% NaCl), suggesting adaptation to hypertonic environments (3, 70). Its obligate aerobic metabolism explains skin but not gut colonization, implying a recent environmental origin rather than long-standing host association (11). Taken together, the physiological profile points toward an environmental saprophyte adapted to oxidative, nutrient-limited conditions (11, 52). Casadevall et al. proposed wetlands and marine environments as candidate ancestral habitats, where brackish conditions select for salt tolerance and sun-exposed shallow waters impose thermal stress, noting geographic overlap between *C. auris* emergence and wetland distributions (3). However, direct environmental isolations remain rare, and human introduction cannot be excluded in cases with positive environmental samples (93–96).

### Hypotheses for pathogenic emergence

Multiple non-exclusive hypotheses aim to explain the transformation from environmental saprophyte to global pathogen, and we refer readers to detailed treatments of these elsewhere (3, 22, 97–99). Despite their diversity, these hypotheses converge on a common theme: anthropogenic environmental change created conditions favoring thermotolerant, stress-resistant fungi, only differing principally in which selective pressures they prioritize. The climate-driven selection hypothesis provides the most parsimonious explanation for the defining puzzle of *C. auris*: its simultaneous, independent emergence on multiple continents. Rising temperatures can directly select for thermotolerance in fungi, as demonstrated experimentally (100). If the most recent common ancestor (MRCA) of modern *C. auris* clades existed within the last 339 years, and individual clades emerged approximately a decade before detection; their origins coincide with accelerating anthropogenic warming (25). If *C. auris* possessed a latent capability to colonize mammals, progressively rising environmental temperatures would have favored thermotolerant genotypes, eventually enabling the breach of the 37°C threshold. Because this selective pressure operates globally, it would act on geographically isolated populations in parallel, explaining the independent emergence of distinct clades across continents.

Several complementary mechanisms likely contributed. Agricultural azole fungicides may have pre-selected for drug resistance in environmental populations before clinical emergence (101), though this alone does not account for resistance to non-azole antifungals, and azole use began decades before *C. auris* appeared without producing comparable emergence in other species (3). More plausibly, agricultural antifungals amplified pre-existing resistance mechanisms that were initially selected through cross-protection conferred by overlapping environmental stress responses. Anthropogenic habitat disruption from wetland drainage, mining, and land-use change may have increased human contact with ancestral *C. auris* populations while simultaneously fragmenting habitats in ways that created population bottlenecks, potentially visible in the low within-clade genetic diversity observed today.

More speculative proposals include avian reservoirs and horizontal gene transfer. The thermotolerance of *C. auris* (up to 47°C [48]) is compatible with avian body temperatures (40–44°C), and migratory birds could in principle explain multi-continental presence (3). However, direct evidence of *C. auris* in wild bird populations is absent, and the genetic distinctiveness of continental clades argues against recent dispersal through a shared avian vector. Horizontal gene transfer from established pathogens such as *C. albicans* cannot be excluded (3, 37, 102, 103), but virulence is typically a complex, polygenic trait that resists simple horizontal acquisition (3); genetic exchange might have fine-tuned existing capabilities rather than created them *de novo*.

### Synthesis: a convergent eco-evolutionary response

The evidence points to *C. auris* having emerged through the combined action of multiple mechanisms: climate-driven selection acting on pre-existing stress-response pathways, with agricultural contamination, habitat disruption, and healthcare transmission amplifying the lineages it favored (summarized in Fig. 1). *C. auris* did not evolve novel pathogenic machinery; it became compatible with mammalian hosts once environmental conditions shifted far enough. Its most recent common ancestor with other fungi predates the industrial era. Thus, the latent genetic potential for thermotolerance existed before warming made it advantageous. Crucially, conserved stress-response pathways, cross-protection, and phenotypic plasticity are fundamental features of fungal biology. If one environmental fungus can breach mammalian thermal defenses by activating conserved systems, the thermal barrier is more permeable than assumed. The question broadens from whether climate change will enable more fungi to cross, to how many, how soon, and if we can predict which ones.

## BROADER IMPLICATIONS: BEYOND *C. AURIS*

The evolutionary trajectory of *C. auris* represents more than the emergence of a single problematic pathogen. It is a case study in how systematic environmental change shifts the selective landscape for an entire kingdom. We face not an isolated threat but a fundamental shift in the selective landscape that could generate numerous *C. auris*-equivalent pathogens from the vast reservoir of environmental fungi.

### The scale of the problem

Only a few hundred of an estimated 3–5 million fungal species infect humans, and most only opportunistically (104, 105). The thermal barrier accounts for this scarcity. As it erodes, the ratio will shift, and even a deliberately conservative calculation highlights the consequences. Suppose climate change confers thermotolerance on 0.01% of 4.3 million species. That yields 430 species crossing the thermal threshold. Even if 1% of them become opportunistic pathogens, we face at least four novel pathogenic fungi. Conserved stress-response pathways and cross-protection are widespread, so this figure is a lower bound, and the true number may plausibly be higher.

### Parallel evolution across fungal lineages

*C. auris* is not an isolated anomaly. Multiple fungal species have recently expanded host ranges, enhanced thermotolerance, or emerged as novel pathogens, suggesting that environmental change exerts parallel selective pressures across diverse fungal lineages.

*Cryptococcus gattii* has expanded from tropical regions into temperate zones, causing infections in previously unaffected areas (11). *Emergomyces africanus* emerged in South Africa as a cause of disseminated mycosis in immunocompromised individuals (106). *Batrachochytrium salamandrivorans* has devastated amphibian populations globally (1, 11). *Aspergillus fumigatus*, among the most prevalent fungal pathogens, is thought to have developed azole resistance through agricultural fungicide exposure (107–109). The related *Candida haemulonii* complex shows increasing clinical relevance despite historically lacking thermotolerance (3, 39), and *Candida parapsilosis* has developed fluconazole resistance with a growing global case burden (110, 111). *Trichophyton indotineae*, first classified in 2020 (112), is now the dominant cause of dermatophytosis (113), which affects 20–25% of the global population, with increasing drug resistance reported from India (107).

Perhaps most striking is the recent case of *Rhodosporidiobolus fluvialis*, a marine yeast identified as the cause of fungemia in two unrelated patients in China. Incubation at 37°C induced rapid mutagenesis in *R. fluvialis*, generating phenotypes with increased virulence and resistance to fluconazole and caspofungin, a process attributed to reactive oxygen species accumulation during heat stress (114). This temperature-induced mutagenesis represents a mechanism through which rising environmental temperatures could drive similar evolutionary transitions in other species.

These examples share common features: recent emergence or range expansion, association with environmental change, and exploitation of previously inaccessible thermal or immunological niches. The pattern is unlikely to represent a series of independent evolutionary accidents; rather, it reflects the predictable outcome of environmental change systematically selecting for adaptations across fungal communities.

### Structural inequality and disproportionate burden

The burden of emerging fungal pathogens falls unevenly. Tropical and subtropical regions face the greatest risk (baseline temperatures already near mammalian body temperature, larger fungal reservoirs, and more severe climate impacts) yet possess the least capacity to respond (1, 11, 115). Molecular diagnostics to identify *C. auris* are often unavailable in resource-limited settings, effective antifungals may be unaffordable, and infection control strains already-stretched systems. The hospitals most likely to encounter these pathogens are thus the least equipped to contain them.

Socioeconomic conditions further compound disease risk (52, 116). Immunocompromised populations, including individuals with HIV/AIDS, cancer patients, and transplant recipients, face the highest susceptibility yet are disproportionately represented in low-income communities with limited healthcare access (52, 116). Malnutrition, endemic infections, and chronic diseases further compromise immunity, expanding the pool of vulnerable individuals, while agricultural workers, miners, and others who disturb soil environments face increased exposure, often with inadequate protection (117).

The same regions experiencing the greatest anthropogenic environmental disruption, where intensive agricultural fungicide use and industrial activities impose novel selective pressures on fungal communities, may serve as evolutionary pools for emerging pathogens while lacking the resources to monitor or respond to threats they generate (3). Global health infrastructure has historically prioritized viral and bacterial threats. While this is beginning to shift, the legacy of neglect manifests as limited surveillance, inadequate diagnostic capacity, and insufficient investment in antifungal development for diseases that primarily affect low-income populations (52).

### The limits of traditional control

Traditional approaches to emerging infectious diseases, such as surveillance, containment, and treatment, face fundamental challenges when applied to fungal pathogens (52). Unlike bacterial or viral diseases spreading through defined transmission chains, fungal infections arise primarily from environmental exposure, and controlling environmental reservoirs is effectively impossible: we cannot sterilize soil, eliminate airborne spores, or prevent human contact with the fungal world (117).

*C. auris* illustrates these limitations starkly despite rigorous cleaning protocols (11, 39). If a known pathogen cannot be controlled even within the relatively contained hospital environment, our capacity to manage multiple emerging species across diverse environmental reservoirs is severely limited.

The treatment-resistance cycle creates an additional challenge without clear resolution (116). Widespread antifungal use selects for resistance, but withholding treatment condemns infected patients to high mortality. Unlike antibacterial stewardship programs, which can sometimes reverse resistance trends by reducing selection pressure, antifungal resistance can arise from environmental selection independent of clinical drug use (3, 52, 101). The rate of resistance emergence has historically outpaced the development of new antifungal agents (101), raising the prospect of encountering pathogens that arrive already resistant, leaving clinicians without effective interventions from the moment of emergence.

These limitations underscore a central point: if reactive control strategies are inadequate even for known pathogens, they will be overwhelmed by the emergence of multiple novel threats.

## FUTURE DIRECTIONS: TOWARD PREDICTIVE MYCOLOGY

The emergence of *C. auris* reveals a pattern: conserved molecular mechanisms, climate-driven selection, and human-mediated dispersal converging to breach mammalian thermal defenses, a pattern that is likely to recur across fungal lineages. Responding effectively requires transforming our approach from reactive crisis management to predictive mycology: integrating evolutionary theory, ecological surveillance, and computational modeling to forecast which fungi pose the greatest threats, where they will emerge, and how to intervene before they do.

### Environmental surveillance and predictive modeling

Mathematical models enable exploration of processes too slow to observe directly, span scales from molecules to ecosystems, and test interventions before deployment. Computational models of heat shock responses in *Candida albicans* have successfully predicted pathogen behavior under clinically relevant conditions that are difficult to study experimentally (118). Laboratory evolution demonstrates that fungi can rapidly evolve thermotolerance through multiple genetic routes (100, 119), and population genetic approaches pioneered in plant pathogen research, using genome scans to identify signatures of adaptation, demonstrate feasibility for predicting evolutionary responses to environmental change (120, 121).

The critical limitation is the absence of ancestral population data to parameterize these models (11). We observe clinical isolates after their emergence but miss the selective episodes that precede detection. This necessitates prospective environmental surveillance: sampling environmental fungi now, characterizing thermal-tolerance distributions, quantifying genetic variation, and measuring selection coefficients under warming scenarios to provide the empirical foundation for evolutionary forecasting. Metagenomic sequencing of hospital air, high-risk soils, and agricultural settings where antifungal exposure selects for resistance would create early warning systems for emerging thermotolerant fungi (122).

Ecological niche modeling demonstrates feasibility at geographic scales. Climate projections predict substantial range expansions for soil-borne pathogens such as *Coccidioides*, with infections potentially increasing 50% as endemic regions shift (122). Genomic offset modeling has forecast distribution shifts in forest fungal pathogens (123) successfully. Extended to human pathogens, such approaches could map regions where environmental conditions will become permissive for thermotolerant fungi, overlaying disease risk onto population distributions to identify vulnerable communities before outbreaks occur.

The ultimate goal is integration across scales: linking molecular mechanisms to population dynamics to epidemiological outcomes. Agent-based models provide computational scaffolding for this integration, simulating individual fungal genotypes competing under climate selection and occasionally dispersing into healthcare settings, where epidemiological dynamics determine outbreak success. The framework exists; implementation requires sustained interdisciplinary collaboration and robust data infrastructure.

### Technological development and capacity building

Prediction alone is insufficient without the tools to act on it. Definitive identification of *C. auris* currently depends on MALDI-TOF mass spectrometry with a curated reference database or on sequencing of the ribosomal ITS and D1/D2 regions, while rapid screening relies on species-specific real-time PCR (19, 124). Standard biochemical panels misidentify the species, and both mass spectrometry and sequencing need capital equipment and reference databases that many hospitals lack, so definitive identification can take weeks (11). Such delays can prove fatal for critically ill patients. Closing this gap means more than shipping instruments. It would entail placing validated PCR assays and reference spectra in regional laboratories, supplying the reagents and controls they consume, training staff to run and interpret them, and funding this durably rather than once. This is what we intend by technology transfer and capacity building.

Drug development must move beyond single-target paradigms that inevitably lead to resistance. Conserved stress-response pathways, such as Hog1, represent attractive targets for broad-spectrum antifungals effective against diverse emerging pathogens (70, 71). Host-directed therapies that enhance immunity or disrupt virulence mechanisms could provide antifungal activity without selecting for resistance (39). Novel agents currently in clinical trials may offer temporary relief (11), but given the speed at which resistance evolves, continuous development pipelines rather than one-off drug approvals are essential.

Critically, the regions facing the greatest risk possess the least capacity to respond (122, 125). This effort must therefore prioritize the tropical and low-income settings that carry the greatest risk and the least capacity (122, 125), and it should be led by local researchers rather than imposed from outside; their knowledge of local ecology, transmission, and healthcare constraints will inform sustainable solutions. Bioinformatic tools for identifying essential gene sets directly in organisms of interest, rather than extrapolating from model species, represent a key priority (39, 57).

An effective response, therefore, demands coordination among medical mycology, evolutionary biology, climate science, and public health. The infrastructure for pathogen surveillance exists: influenza forecasting and vaccine strain selection demonstrate that pathogen prediction works when adequately resourced. However, equivalent systems for fungal pathogens require sustained investment and cross-sectoral coordination between medical, agricultural, and environmental domains.

Pre-determining likely candidate species would enable informed, anticipatory screening and expedited diagnostics, gaining crucial time for patients, healthcare facilities, and researchers. Open-access repositories for fungal genomes, phenotypes, and clinical outcomes would provide the raw material for computational prediction, while population genomic analyses integrating both polymorphism and divergence data can disentangle the types of selection acting along genomes and identify functionally important loci (126). Understanding how evolutionary potential is maintained in pathogen populations despite strong directional selection requires close integration of evolutionary genomics with experimental studies (121).

## CONCLUSION

The emergence of *C. auris* forces a recognition that mammals’ historical freedom from fungal disease was not a permanent feature of our biology, but a contingent consequence of environmental conditions that are now changing. We evolved in a world where temperature separated us from most fungi; that world is disappearing. Ectothermic vertebrates already experience fungal disease at rates that would be catastrophic for human populations; the thermal immunity that distinguishes mammals from amphibians, reptiles, and fish is being systematically eroded. The future of mammalian immunity revolves around one central question: Why did *C. auris* in particular emerge when others did not? The strategies it uses are conserved and widespread, so we should expect a rise in infections caused by other fungi, especially related *Candida* species. Candidiasis caused by other *Candida* spp. is indeed rising (39, 110, 111, 127), which, alongside the cases in “Parallel evolution across fungal lineages” above, suggests we are observing the early stages of emergence. These species are less studied than *C. auris*, and their records carry the same misidentification and reclassification problems. With the *Candida* phylogeny unresolved and case documentation patchy, the evidence does not yet support definitive conclusions about which species will establish next.

A pragmatic research agenda emerges from this analysis. In the near term, coordinated environmental sampling campaigns targeting hypothesized reservoir habitats such as wetlands, coastal zones, and agricultural areas should seek to isolate thermotolerant fungi before clinical emergence, while experimental evolution studies across diverse species quantify how rapidly thermotolerance evolves and identify the conserved genetic mechanisms involved (100, 119). In the medium term, ecological niche models predicting geographic risk must be developed and validated (122, 123), novel antifungals advanced through clinical trials (11), and mycological capacity built in the high-risk regions that need it most. In the long term, the goal is a functioning system of predictive mycology in which environmental surveillance feeds real-time data into models that generate probabilistic forecasts of emergence, guiding targeted interventions before outbreaks occur. This vision is ambitious but feasible, and the alternative, accepting repeated crises as thermotolerant, multidrug-resistant pathogens emerge one after another, is untenable.

The promise of predictive mycology lies not in eliminating uncertainty but in transforming how we engage with it. Rather than waiting for the next pathogen to appear, we can monitor the evolutionary landscape, identify species approaching thermal thresholds, and intervene before they cross over (1, 115). This requires treating fungal disease emergence not as a series of independent shocks but as a predictable consequence of systematic environmental change, one that theoretical biology, integrated with empirical surveillance and coordinated global action, can help us navigate.

## Data Availability

The data underlying Fig. 2 are available at https://github.com/tecoevo/c_auris, and the review by Jones et al. (128) has proved invaluable in compiling these data.
